# Comparing shades of darkness: trolling victims’ experiences on social media vs. online gaming

**DOI:** 10.3389/fpsyg.2023.1163244

**Published:** 2023-08-22

**Authors:** Christine L. Cook, Simon Y.-C. Tang, Jih-Hsuan Tammy Lin

**Affiliations:** ^1^International College of Innovation, National Chengchi University, Taipei, Taiwan; ^2^College of Communication, National Chengchi University, Taipei, Taiwan; ^3^Department of Advertising, National Chengchi University, Taipei, Taiwan; ^4^Taiwan Institute for Governance and Communication Research, Taipei, Taiwan

**Keywords:** trolling, toxicity, online gaming, social media, victimization

## Abstract

Although there is ample literature available on toxicity in games, as there is regarding trolling on social media, there are few to no cross-platform studies on toxicity and trolling. In other words, the extant literature focuses on one platform at a time instead of comparing and contrasting them. The present work aims to rectify this gap by analyzing interviews from a larger study of 22 self-proclaimed victims of in-game trolling to not only determine whether social media or gaming communities are considered more toxic but also to explore how definitions of the word ‘trolling’ change depending on the platform in question. We found that while definitions of in-game trolling behavior focused on behavioral styles of trolling (e.g., throwing one’s avatar into enemy fire to disadvantage one’s team, and blocking other players’ avatars’ movement), social media trolling is defined by more sinister actions such as misinformation spreading and ‘canceling’ other users. We also found that gaming is perceived as generally more toxic than social media, often due to company policies or lack thereof. Practical and theoretical implications for the study of toxicity in all online communities – gaming or social-media based – are discussed.

## Introduction

Since its inception, the internet has been used for social interactions both benign and malicious (e.g., Herring et al., 2002; Graham, 2019). At the outset, this took a largely textual form, with the earliest ‘hate raids’ – where users from one community invade another community’s space to annoy or attack said community – taking place on Usenet (Graham, 2019) and later on modern internet fora (Herring et al., 2002), and with the first cases of trolling happening in text-based virtual worlds called multi-user dungeons or ‘MUDs’ (Dibbell, 1993). Back then, games and social media were not so different; these all consisted of people communicating with one another through text shared via cyberspace. Over time, however, social media and gaming began to differentiate themselves from one another and develop their own unique subcultures as they developed new affordances and mechanics (e.g., Baird, 2022; Mitchell-Church and Thambusamy, 2022). Today, few people would confuse Fortnite and Facebook, for instance, but both share the good and bad communication that their Usenet and AOL forebears once did (e.g., Hannan, 2018; Hilvert-Bruce and Neill, 2020). However, there are few studies that directly compare and contrast the kinds of communication that happen on these platforms.

Trolling – a form of negatively perceived communication that exploits fellow netizens using website, game, or chat mechanics online (Cook et al., 2021) – can take many forms: verbal, non-verbal (often called behavioral in extant literature; see Cook et al., 2018), platform-specific, or more general. For example, while flaming (extensive use of profanity and/or personal insults) and spamming (repeatedly sending the same message non-stop) can occur on both social media platforms and in games using chat functionalities, stealing someone’s kill bonus or blocking the movement of another person’s avatar is only possible in certain types of games (see Kowert and Cook, 2022 for a more complete list of trolling behaviors in games). Trolling behavior has been found across most major platforms, including Twitter, Facebook, and YouTube (McCosker, 2014; Craker and March, 2016; Synnott et al., 2017), and nearly all forms of multiplayer gaming have some kind of trolling within them, though team-based multiplayer games such as League of Legends are notable for their extensive research on the topic (Coyne et al., 2009; de Seta, 2013; Blackburn and Kwak, 2014; Arjoranta and Siitonen, 2018; Kou, 2020). Research has also shown that the more severe forms of trolling – including but not limited to repeated harassment and identity-based insults – can lead to consequences comparable to those of cyberbullying, including heightened anxiety, depression, and withdrawal (Gray et al., 2016; Campbell, 2017; Fox and Tang, 2017). Essentially, we know that independent of one another, trolling occurs on both social media and gaming platforms. What we do not know is which is perceived as being more dangerous and/or toxic to its users.

The present study’s aim is, therefore, to look at trolling on both gaming and social media platforms. More specifically, we spoke to people who have self-identified as victims of trolling in games and asked them (1) how their understanding of trolling differs between gaming and social media, (2) which behaviors constitute trolling on which platforms, and (3) how the perceived prevalence of trolling differs between social media and gaming platforms. From a theoretical point of view, this will add nuance to our understanding of what trolling is and how the platform shapes our understanding of the phenomenon. Practically, it will also enlighten policy-makers and platform owners and developers as to which kinds of behaviors they need to target and where best to protect their users.

## Theoretical background

A recurring theme in trolling literature is the struggle to establish a single definition of trolling itself (Hardaker, 2010; Cook et al., 2021). While one definition might emphasize deviance (e.g., Fichman and Sanfilippo, 2014), another will focus on the idea of destruction or discord (e.g., Buckels et al., 2014). One idea that has been posited as an explanation for these different emphases is that these articles are all coming from different disciplines and different populations (Cook, 2021). This could explain, for instance, why Thacker and Griffiths (2012) – social psychologists working in a gaming psychology space – emphasized instrumentality in their definition as game players are often literally using game mechanics to achieve their trolling aims. Synnott et al. (2017), by contrast, emphasize the offensiveness of trolling as they focus on the targeted social media trolling of a single family and the criminality of this trolling and harassment. By looking at different people in different spaces, it seems natural that certain elements of trolling would become more or less prominent depending on the researcher’s perspective.

It is important here to note that, within the realm of games research specifically, there is also considerable discussion about the overlap of the term “trolling” with other similar terms, such as grief play and toxicity. Each of these terms comes with its own distinct literature, and so, before we move on to trolling on social media, we will briefly cover each of these terms and how they relate to one another. Toxicity is specifically the behaviors that are intentionally malicious and hurt other players (e.g., Boudreau, 2019; Kordyaka et al., 2020; Beres et al., 2021). This is often the kind of trolling discussed in media covering trolling and is akin to harassment, abusive communication, and flaming (Beres et al., 2021). It is also important to note that marginalized game players are more often than not the targets of toxicity (Boudreau, 2022). All of these abusive behaviors have, at one time or another, also been listed as forms of trolling (see Herring et al., 2002; Kowert and Cook, 2022). In the present article, we therefore consider toxicity to be a subset of trolling more broadly: a kind of trolling that has caused harm (since we are talking to victims instead of trolls, we cannot confirm whether or not this harm was intentional). It should be noted that some of the behaviors that previous works have grouped into trolling are not universally agreed upon to constitute trolling (sexism, racism, and transphobia, etc.), and these often have their own rich literature. However, in the interest of avoiding the invalidation of targets’ experiences of what they have determined to be trolling, we have included these as a part of our broader conceptualization of what trolling is in the present work.

Grief play is extremely similar to trolling, with Stenros (2015) describing it as “a collection of disruptive activities that are usually discussed as problematic, or something to be eliminated” (p. 176) and later as “an undesirable side effect of multiplayer games” (p. 177). These definitions also neatly correspond to existing definitions of trolling coming out of gaming spaces (see Thacker and Griffiths, 2012; Cook et al., 2021). Like the existing work on trolling (e.g., Cook et al., 2018), work on grief play is careful to emphasize that there is an element of playfulness, even if it is at the expense of others (Paul et al., 2015; Stenros, 2015). Grief play (also called “griefing”) is also not limited to a specific set of “griefers” but is rather a type of play that many game players engage in from time to time (Stenros, 2015). Much of what is described as grief play has also been called either toxicity (see Beres et al., 2021) or trolling (see Thacker and Griffiths, 2012; Kowert and Cook, 2022) at some point, including scamming and ninja looting, which is the act of stealing in-game items from a defeated enemy that were not designated as belonging to the stealer in a group setting (Foo and Koivisto, 2004). Unlike toxicity, however, there is no specific implication that griefing is intentionally malicious; it is transgressive (Boudreau, 2022), but it may not necessitate the explicit intention to cause harm to others (Foo and Koivisto, 2004; Stenros, 2015). Therefore, when, in the present article, we examine “trolling” and “trolling victims,” we are conceptualizing this as the kind of toxic trolling or grief play that causes harm to another gamer, be that harm minimal or more extreme.

However, just as Thacker and Griffiths (2012) do not provide the only gaming-based definition of trolling (e.g., Wright, 2019; Kowert, 2020), neither are Synnott et al. (2017) the only researchers to try and define the phenomenon in some way via social media (e.g., McCosker, 2014; March et al., 2017). Even within a single platform or context, trolling definitions vary considerably. For instance, while Thacker and Griffiths (2012) define trolling as “an act of intentionally provoking and/or antagonizing users in an online environment that creates an often desirable, sometimes predictable, outcome for the troll” (p. 18), Wright (2019) defines it as “creating arguments by upsetting people either through talking about or posting provocative or off-topic messages in online communities” (p. 605). Both of these research groups studied games and game players and covered a variety of games, although Wright (2019) had a notable focus on console gaming that was not present in Thacker and Griffiths’ (2012) work. Altogether, this would suggest that platform – even within the same category of users – plays a role in how we understand trolling and toxicity. In the interest of further narrowing down the definition debate, the present work will take a single population – thereby removing the inherent individual differences present when comparing studies using both different platforms and different samples per platform – and ask them about trolling on multiple platforms to see how, if at all, their understanding of trolling differs according to platform. More specifically, we aim to address the following research question:

RQ1: How, if at all, does game players’ understanding of trolling differ depending on the platform on which the trolling behavior occurs?

### Trolling behaviors and prevalence

Another potential difference between trolling on gaming and social media platforms could be the trolls’ chosen methodology. In games, research has shown that although trolls will engage in their fair share of verbal trolling (see Thacker and Griffiths, 2012; Wright, 2019), another major component is what the literature calls behavioral trolling (see Cook et al., 2018; Kowert and Cook, 2022), in which game players exploit the mechanics of the game itself to troll their targets without using a chat function. Examples of this could be purposely throwing one’s avatar into the enemy to give the enemy the benefits of a kill (often called ‘feeding’) or using one’s avatar to block the movement of a teammate (often called ‘body-blocking’). Although this kind of exploitation does occur outside of the gaming sphere – one can think of Rickrolling, in which a person is bait and switched on YouTube when they expect one type of video and is treated instead to the music video of Rick Astley’s 1987 hit “Never gonna give you up” (Baudry and Monperrus, 2022) – it appears less frequently in extant literature. Instead, literature focusing on social media tends to focus on the verbal elements of trolling: misinformation and the weaponization of information (Kargar and Rauchfleisch, 2019; Kirkwood et al., 2019), nasty comments on social media posts (Lopes and Yu, 2017; Masui, 2019), or making inflammatory posts to provoke others (Navarro-Carrillo et al., 2021). In academic literature, at least, behavioral trolling seems largely relegated to gaming, while verbal trolling – which receives more attention overall – seems more evenly spread across the two platform types (see Cook, 2021). Put in other words, the current state of research would suggest that those who troll in games take advantage of more affordances when engaging in trolling behavior than those who troll using social media.

That said, this could reflect a bias in research more than an actual difference between the platforms. For instance, Paul et al. (2015) point out that, at least in gaming, there is some degree of simple playfulness in trolling, particularly in terms of behavioral trolling (called “griefing” in that article). Some of this pleasure is sadistic, as is often considered the case for trolling outside of games (Buckels et al., 2014), but not all. Cook et al. (2018) also found that gamer trolls sometimes engage in trolling behavior exclusively to form friendships or start up banter within a team setting. However, there is far less research dedicated to this more “fun” type of trolling as from a funding and societal impact perspective, it is less urgent to “solve” playful trolling than it is to deal with the more malicious forms of trolling behavior. Consequently, behavioral trolling, which often falls into this more playful category, is an even less desirable a research topic for those outside of the gaming sphere. Given the limited understanding of trolling prevalence in either space (see Kowert and Cook, 2022), and the general lack of cross-platform studies in trolling research as a whole (see Cook, 2021), it is difficult to gauge what is an artifact of our own interests as researchers and what is representative of a genuine difference in platform. The present work aims to bridge that gap by asking game players about what they consider to be trolling on both social media and gaming platforms and also by inquiring about the prevalence of trolling on social media and in gaming. We will, more precisely, address the following research questions:

RQ2: Which behaviors constitute trolling on social media platforms and gaming platforms, respectively?

RQ3: What is the perceived prevalence of trolling on social media and gaming platforms?

## Methods

### Participants

The present study was part of a larger interview project with trolling victims involving 22 participants (17 men, 3 women, and 2 non-binary) between 18 and 36 years of age (*M* = 23.64, *SD* = 4.33). All but one (who selected Google Meets) participated via Discord. Please see Table 1 for a full demographic breakdown. On average, the participants engaged in 2.73 (*SD* = 1.99) hours of daily gaming, most of which took place in multiplayer games. The most popular genre was multiplayer battle arenas (MOBAs, 17), followed by first-person shooters (FPSs, 8), then massively multiplayer online roleplaying games (MMORPGs, 6); other genres mentioned included, in order of popularity, strategy games (5), mobile games (2), and console games (1). The participants used various social media platforms from 1.5 to 12 h a day (*M* = 4.55, *SD* = 3.53). In order of popularity, these platforms were Twitter (16), Instagram (15), Facebook (14), Discord (12), Reddit (7), TikTok (4), LinkedIn (3), WhatsApp (2), Twitch.tv (2), and Snapchat (2). These game players do not represent any specific strata of society and are as diverse a convenience sample as we were able to recruit via social media advertisements in English, the language of the interviewer. It is important to note that although several cultures are represented within the sample, we are not performing a culture-by-culture analysis due to the limited size of the sample.

**Table 1:** Participant demographics.

	Age	Gender	Residency	Citizenship	Ethnic Minority	LGBTQ+
Participant 1	26	Man	Europe	Europe	No	No
Participant 2	21	Man	South America	South America	No	No
Participant 3	21	Man	North America	North America	No	No
Participant 4	24	Man	Europe	Europe	Yes	No
Participant 5	23	Man	Asia	Asia	Yes	No
Participant 6	18	Man	Asia	Asia	Yes	No
Participant 7	25	Man	North America	North America	No	No
Participant 8	25	Woman	South America	South America	No	No
Participant 9	21	Man	North America	South America	No	No
Participant 10	19	NB*	North America	North America	No	Yes
Participant 11	20	Man	North America	North America	No	No
Participant 12	25	Man	North America	North America	No	No
Participant 13	32	Man	Europe	Asia	Yes	No
Participant 14	18	Man	Oceania	Asia	Yes	No
Participant 15	23	Man	Asia	Asia	Yes	No
Participant 16	20	Man	North America	North America	Yes	No
Participant 17	25	Man	Europe	Europe	No	No
Participant 18	22	Man	North America	North America	Yes	No
Participant 19	25	NB*	North America	Asia	Yes	Yes
Participant 20	36	Man	North America	North America	No	No
Participant 21	28	Woman	North America	North America	Yes	No
Participant 22	23	Woman	Asia	Asia	Yes	No

* = Non-binary.

### Procedure and materials

After receiving their institutional review board’s ethical approval, the authors recruited people who (1) had been trolled at least once before in a gaming context, (2) were at least 18 years of age, and (3) were able to both receive payment and participate in an interview online. This recruitment took place via Twitter and Facebook posts, both of which were shareable by other users. It was a convenient sample. In order to avoid biases toward specific types of trolling, no definition was given to participants at any point in the recruitment or study phases. This also avoided the difficulty of selecting only one of the multiple definitions of trolling (Hardaker, 2010). Participants signed a digital consent form and then scheduled a time slot for the interview with the first author. All but one interview was recorded with the participants’ permission using the Craig bot Discord program (see https://craig.chat/home/); the one participant using Google Meets was recorded with a Google Chrome extension (see https://fireflies.ai/).

As this was part of a larger study, the complete interview protocol will not be addressed here in full (see Appendix A in Supplementary material). Questions were generated *a priori* by the authors based partially on extant work interviewing trolls (Cook et al., 2018), to allow for comparison between how trolls view trolling versus how victims do, and partially on themes that were perceived to have gaps in the existing literature when it comes to our understanding of trolling victims. The questions that concerned the present work were those pertaining to comparisons between social media trolling and online gaming-based trolling. We asked participants to compare definitions of trolling, trolling behaviors, and frequency of trolling across platforms. We also asked the participants if any platforms were doing specifically well or poorly when it came to managing trolls and trolling. However, these interviews were semi-structured, meaning the interviewer was allowed to probe further should a new topic of interest arise naturally throughout the course of the interview. Interviews lasted between 36 and 95 min (*M* = 63.64, *SD* = 15.25). After a verbal debriefing and answering any questions participants had post-interview, they were paid the local equivalent of 15 euros via PayPal (with one exception who received it in cash).

### Analytical strategy

At the beginning of our analyses, all recordings had already been transcribed via Temi.com and reviewed by the second author for quality control. The first author then highlighted all instances in each interview where social media and gaming-based trolling were compared and developed a codebook using a grounded theory approach (see Strauss and Corbin, 1990), meaning all categories were first found in the data proper as opposed to being made a-.

priori. The first two authors then independently coded each interview transcript using this codebook (see Appendix B in Supplementary material for codes) to allow for a systematic comparison of social media and gaming from the participants’ perspective. After coding for each of the three research questions, the two authors met to compare codes. They also discussed any discrepancies in the coding and decided on a final code for each participant response; the completed list of final codes was used to create our results.

## Results

### Comparing social media and gaming trolling definitions

When coding for the definitions of trolling on social media and online games, we found six categories of definitions: (1) intentional antagonism, (2) cancelation (social media only), (3) misrepresentation (social media only), (4) breaching privacy (social media only), (5) not trying to win (gaming only), and (6) playful “messing with”. These are not, however, mutually exclusive; some participants’ definitions included elements of two categories, while five participants failed to define trolling in social media, considering it a game-exclusive phenomenon.

Our first observation was that participants had a much firmer, more coherent understanding of trolling in the gaming context; there was a lot more variety in terms of understandings of what it meant to troll on social media or if it was even possible (see above). In the gaming context, only three of the six possibilities emerged: intentional antagonism, not trying to win, and playful ‘messing with’. Of these, intentional antagonism was the most popular, with 17 of the 22 participants defining in-game trolling in this way for games and 11 defining social media trolling in this way. P6 defined trolling as follows: “I will classify trolling as annoying people … and harming other people … harming their experience … either verbally or using mechanics in the game to annoy other people for fun.” This summarizes the category well; trolling, to these people, is the act of ruining someone’s experience of a game or platform. This was the third most popular definitional category for social media as well. For gaming, however, the next most popular definitional category was “not trying to win the game” (12 out of 22 participants). In this category, the participants stressed actions such as “feeding” the other team by intentionally getting one’s character killed (P14; P15), thereby assisting the other team to the detriment of one’s own team. Finally, three participants (P19; P20; P21) included elements of playfulness in their trolling definitions for both games and social media. For them, trolling is not purely evil as those in the intentional antagonist camp seem to believe but rather “playful, like joking, like messing with others in game” (P19). P20 describes trolling as:

behavior that kind of edges, the cusp of being downright toxic, uh, but is not necessarily toxic per se. It's, it's, it's that gray area that I would liken unto the kid in junior high that kind of wants to be your friend but doesn't want to be uncool. So, they pretend to be your friend and then laugh at you for thinking that you were friends with them.

In short, game players seem to believe that trolling in games is heavily tied to intentionality; whether that intention is malicious or playful, trolling is an action with a specific goal in mind, usually to capture a reaction from the target.

On social media, things are far murkier. In addition to the 11 participants who said that trolling was intentional antagonism, irrespective of platform, there were also some participants (P2; P12) for whom trolling is synonymous with cancel culture, which is the public removal of support from a person, usually due to an apparent moral failing (see Ng, 2020; Cook et al., 2021). It should be noted that cancel culture has its own literature and is not always grouped under the label of trolling. In the case of our participants who mentioned it, the key point that made it trolling for them was the intention to ruin someone’s reputation *in the case where the person was not perceived as deserving of such a punishment.* This changed it from the canceling of a public figure to character assassination via online insults: an aggressive form of trolling. For instance, P2 describes social media trolling as “accusing people of stuff and maybe calling people for their preferences for no reason. Like, if I have an opinion, then you go out and say, ah, your opinion is bad.” However, for others, trolling on social media is more about misrepresentation (5 out of 22 participants), either by spreading misinformation (e.g., P4) or by pretending to support a controversial cause in order to get a rise out of their target (e.g., P11). P17 describes this as “saying one thing and doing the other,” a sort of bait-and-switch technique similar to Rickrolling in the early days of YouTube (see Cook et al., 2018). Finally, P5 equated social media trolling to doxing (i.e., revealing someone else’s personal, private information online without their permission), saying that trolling on social media is “[breaching] someone’s privacy, like they stalk your account and then like [post] your photo.”

However, what is perhaps the most interesting aspect is that our participants seemed to find trolling on social media to be more playful than in gaming, with 9 of our 22 participants including an element of playfulness in their definition and 4 of those (P4, P6, P8, and P20) defining social media trolling as a purely playful behavior. P8 describes it as “making bad jokes,” while P20 explains that on social media, “it’s a bit more nuanced … because the rules of society are much more intricate and complicated than, than the rules of a video game,” but concludes that trolling is “infuriating … [but] it [is] hilarious to the troll … and it [is] moderately amusing to some of us that [witness] it unfold.” Essentially, it would appear that social media trolling is more varied in its intent than in-game trolling, or at the very least, this is how victims seem to perceive it.

### Comparing social media and online game trolling behaviors

The summary of our results is presented in Table 2. We can see that what most users consider to be trolling differs significantly between social media and online games. On social media, trolling seems to take an especially personal and verbal dimension, with misinformation, teasing, and personal insults being the top three most common ways people seem to troll. In games, however, the dominant view is that trolling is something behavioral; common types of behavioral trolling mentioned include “inting” or “running it down”, which refer to getting one’s own character killed on purpose in order to disadvantage one’s team and help the enemy team in games, and also selecting off-meta picks, which is when users decide to choose a character that is ill fitted to their role in team games, thereby disadvantaging their team. This is consistent with the extant literature (e.g., Synnott et al., 2017; Kowert and Cook, 2022). That said, profanity/flaming and personal insults were also found to be popular in gaming.

**Table 2:** Number of times each type of trolling behavior was mentioned per platform.

Trolling behavior	Explanation	Social media	Online games
Profanity/Flaming	Use of foul language or typing in all caps to simulate yelling.	7	6
Doxing	Stealing and spreading personal information from another user.	3	1
Verbal Harassment	Repeatedly attacking a person verbally.	5	2
Misinformation	Willfully spreading information the person knows to be false.	13	4
Provocation	Speaking with the sole goal of eliciting an overreaction from the target.	4	3
Behavioral Trolling	Inhibiting others’ goal acquisition through using platform affordances.	0	20
Making fun of or Teasing	Playfully insulting or “messing with” others.	10	2
Spamming	Repeatedly typing the same thing into a chat box or saying the same thing verbally over voice chat.	3	1
Personal Insults	Attacking someone verbally based on an identity marker (LGBTQ status, religious affiliation, etc.).	9	9
Hacking	Using cheats or otherwise exploiting the programming of a platform in an illegal way.	1	4
Memes	Spreading gifs or images as a way to annoy or make others laugh.	2	1

Originally, misinformation and lying were separate categories, but they were merged at the final coding.

What seems to separate social media trolling from gaming trolling, at least according to trolling victims, is its core motivation. If we look at the most popular types of in-game trolling, they are things that previous literature has rooted in frustration (e.g., Cook et al., 2018; Cook, 2019; Kowert and Cook, 2022). We can see the same thoughts expressed by P3, who describes trolling in League of Legends as follows:

Well, I mean, there's a lot of times where trolling is just about, you know, someone, like, slightly annoyed you while you were playing a game, um, they took your kill or something like that. These used to be super common, um, like a few years back, especially in, in, uh, maybe lower levels of play and league. But, like, for example, if, uh, your support takes a kill, then you just start trolling, um, just out spite really. There's no, like, rhyme or reason to it.

Essentially, when things stop going one’s way – when one’s goals are frustrated by another player – this catalyzes a trolling response in games. Social media trolling, by contrast, does not seem to be borne primarily of frustration but rather out of boredom. P4, for instance, gives the example of “[joining] a flat Earth society as a joke and then [perpetuating] that idea ironically … that would be a troll.” When considering personal insults, however, these motivations seem to overlap, with many participants mentioning racism and sexism in particular being prevalent on both gaming and social media platforms (e.g., P8-10) and participants from some regions stressing religious targeting (P5). We therefore have to be careful not to keep these two motivations as mutually exclusive and unique to each platform while recognizing that one motivation would seem to predominate over the other depending on the environment.

### Comparing toxicity levels across games and social media platforms

To answer our final research question, we wanted to compare social media platforms and games to see which was perceived as more filled with trolls. Since so few of our participants defined trolling in a playful way (P19, P20, and P21 in games only), it would seem as though trolling is still largely seen as a toxic behavior to some degree, irrespective of platform (see Kowert and Cook, 2022, for a discussion of toxicity vs. trolling). It should be noted that eight of our participants did not specify that either social media as a whole or gaming as a whole was more toxic, though they did talk about reasons why individual platforms were more or less toxic. That said, of those who did specify one or the other as being the most toxic, ten (P2; P9; P11-12; P15-17; P19-20; P22) thought games were more toxic, and four (P1; P13; P18; P21) thought social media was more toxic. In terms of individual platforms, the game that was most frequently mentioned as having a toxic community was League of Legends, while the social media platform that was most frequently mentioned as being toxic was Twitter. Genshin Impact and Reddit, by contrast, were known for their friendliness and lack of trolls.

When probed further, the participants gave a variety of reasons for their choices. These reasons fell into three broad categories: features of the company (Company policies: 11, Content moderation style: 10, Moderation tools available: 13), features of the platform (Community norms: 10, Competition or lack thereof: 6, Anonymity: 8, Affordances: 9), and features of society (Racism/Sexism: 3). One participant was unable to give any reasons for their opinion regarding the toxicity of social media and gaming platforms (P9). Many participants brought up the company policies regarding punishment of trolls as a major source of grief; P18 had this to say about how companies deal with trolling in games:

A lot of the times it's 1% of the people who contribute 50% of the trolling or whatever. I don't think that there is, like, it is to the point where if you were to remove all the trolls from the game or anyone who, who has trolled more than once you'd have no players. I think it's more so take these instances where it is blatantly obvious, where it's actively ruining more than just one game's worth of pain. And, like, you can sort of sniff out the, the real culprits of everything and maybe actually taking, making disciplinary action on them is something that can be take, taken into account.

Still, others focused on content moderation styles of certain platforms, with P20 praising Reddit’s moderators for “[taking] care of [trolls] when they are too prevalent” and P12 asking that Riot Games “bring back a form of the tribunal,” which was a player-based moderation system that existed in the past in League of Legends but has since been largely replaced with automatic tools such as chat filters. In essence, people often blame the companies that run the platforms and their poor decision-making when it comes to policies to punish trolls for toxicity levels today.

However, almost equally important were features of the platforms themselves and, especially, the community norms that develop on these platforms, particularly in light of anonymity. P1 explains that “a lack of physical presence … means that people disconnect the human being they are talking to from the interaction, which makes things very comfortable and … normalizes behavior that would not necessarily be normalized in a real-world setting.” This is almost an exact definition of moral disengagement in the style of Bandura et al. (1996); the anonymity of the platform allows game players to ‘other’ other players and shut off their moral compass, so to speak. P10, by contrast, emphasizes how a good reporting system can neutralize trolling:

Like, if somebody's been being toxic and chat, you can report them. And, usually, they will do something about, like, they actually are pretty good at doing something about it. If you report them for toxicity, uh, and, uh, stuff, kind of simple, like, if they, if they have a report system and actually look at reports, that's a good enough system for me.

In sum, platforms that are lower in anonymity, whose report functions are kept up to date and regularly addressed, and whose companies punish trolls for their actions are the platforms people want to use. Transparency here is key for both social media and gaming platforms alike. Platforms that fail to keep these things in mind not only turn away existing users but can develop a reputation that will ward off new clients.

## Discussion

In the present study, our first aim was to compare how victims defined trolling on social media and how they defined it in the context of online gaming. Although there were elements that were the same across platforms, we did find differences in their understandings. The definitional categories provided for online gaming trolling – intentional antagonism, not trying to win, and playful ‘messing with’ – largely correspond to what has already been found in the extant literature on game-based trolling (see Cook et al., 2018), which is to be expected. However, the more varied results of trolling on social media better reflect the diversity of trolling literature as a whole. There is no one conception of trolling as malicious online behavior; rather, trolling can be as calculated as the act of ‘canceling’ associated with cancel culture (Ng, 2020; Cook et al., 2021) or as benign as gentle teasing of a friend. In other words, while the concept of trolling seems to be quite concrete and commonly understood within the gaming context, trolling on social media seems to be more fluid, albeit primarily verbal (spreading misinformation, flaming, etc.). Taken altogether, these results would suggest that part of the difficulty when it comes to unifying trolling research under a single definition is the issue of platform and how people understand trolling in different spaces.

Our second goal was to capture more fine-grained detail regarding which behaviors were considered to be trolling on social media and which were considered trolling in games. The complete findings are presented in Table 2; once more, though there was overlap, we did find distinguishing trolling types that were more prominent on either social media or in online games. The exploitation of programming and affordances for trolling purposes – called behavioral trolling here in accordance with the extant literature (see Cook et al., 2018) – seems to be something that people consider to be game specific, for instance, while misinformation seems to be mostly a social media problem. The latter stands in direct contrast to other trolling works that describe misinformation as one of the key ways veteran players troll newbies in games (Thacker and Griffiths, 2012; Cook et al., 2018). This could reflect several different aspects, all of which would require further research to confirm or deny.

Firstly, it could represent a shift in trolling trends. It has been several years now since Cook et al. (2018) and Thacker and Griffiths (2012) talked to gaming trolls, and it is possible that the more veteran players who employed these tactics have since ‘aged out’, so to speak, of trolling. Second, it could represent a shift in game players’ collective understanding of trolling. Perhaps, the game players of today no longer consider the trickster archetype of the past (see Herring et al., 2002; Thacker and Griffiths, 2012) as the primary form of trolling, instead associating it with more malicious actions such as flaming and behavioral trolling, as indicated by our results. It could be an effect of the traditional media’s current focus on misinformation and combatting misinformation on social media (Gross, 2023; World Health Organization, 2023). Although we did not specifically use the word misinformation in our questions (see Appendix A in Supplementary material), it is still possible that due to their following news on misinformation and disinformation, the participants automatically associate it with social media, simply not thinking of lying to a gamer about what level a monster is as a form of trolling. Finally, it could be an artifact of our limited sample. The majority of our game players are MOBA players, meaning they are making split-second decisions regularly about different plays to make as a team. This puts a lot of emphasis on game mechanics, while MMORPG players, for instance, have more time to talk to one another. Further research with a more varied sample could determine which explanation best suits the gaming population as a whole.

Finally, we wanted to see which platforms were perceived as being more toxic (i.e., included more malicious trolling) and why. On the whole, most participants found online games to be more toxic than social media, but this finding must be contextualized in terms of the participant pool; most of our participants were relatively heavy game players, especially compared to their social media usage, so there could be an effect of time and usage creating this result in part. That said, most participants were able to give clear reasons for their opinions. A major factor in the participants’ opinions regarding toxicity levels was content moderation practices. They were appreciative of automatic tools such as chat filters and automatic detection of profanity on voice chat but stressed that these are not a replacement for human content moderators. There was no clear winner in terms of whether paid or volunteer moderators were considered superior, but there was a definite cry for human moderation of some form. Gaming companies such as Riot Games and Valve were especially criticized for having too little human intervention. Social media companies were also generally perceived as being better at doling out punishments to toxic offenders, unlike most games, whose reporting functions were derided as useless by many of our participants. In other words, game players seem to have the impression that social media is a more controlled environment, while online games are more of a cyberspace Wild West.

However, our results have also highlighted the strong moral undercurrent that seems to apply to all forms of trolling. This concept of intentionality that is so critical to defining something as trolling, at least according to trolls (see Cook et al., 2018) and victims, fundamentally implies a moral choice and that trolling is a moral failing. One of our participants even explained trolling as a normalization of deviant behavior due to anonymity, essentially citing moral disengagement theory (Bandura et al., 1996) as the explanation for trolling online, irrespective of platform. Gaming research is no stranger to morality as single-player games often require players to make choices in the face of moral dilemmas (Joeckel et al., 2012). Joeckel et al. (2012), when examining this question, found that moral salience was an important factor in determining what kind of choice players would make when forced to make a choice: the more salient morality was in the environment/narrative, the less likely one was going to violate moral standards. Does the same thing happen when the moral choice is not forced but, rather, deals with real people and real interactions? On social media, when someone acts against the established social norms, people feel the need to process this negative emotion and behave accordingly, often with punishment for the violator, according to expectancy violation theory (Burgoon and Jones, 1976). Would increasing the moral salience have the same effects across these platforms? More cross-platform research involving moral theories needs to be conducted to find out.

It is also worth connecting the present work to the broader world of media studies. For instance, the concept of stickiness in platform economies could play a role in how platforms differ from one another trolling-wise. User stickiness refers to how often users return to a platform for use (Xu et al., 2018); it is also a key goal in modern platform economics (Laczko et al., 2019; Rong et al., 2019). The stickier a platform is, the more ads and/or products they can theoretically sell using that platform. In their study, Xu et al. (2018) suggest that platforms can enhance stickiness through improving content quality and system quality while encouraging user participation. However, studies such as that of Synnott et al. (2017) demonstrate that user stickiness can be achieved by quality conflict as much as by quality content, thus incentivizing platforms to increase trolling and toxicity rates to increase user stickiness. How much the upper management of different platforms adhere to this principle could theoretically affect perceived toxicity and/or trolling rates on said platforms.

Researchers have also documented how trolling has been used as a political tool to sway opinions and/or discredit ideologies or political figures (Phillips, 2011; Akhtar and Morrison, 2019; Kargar and Rauchfleisch, 2019). This also connects back to our participants who considered canceling as a form of trolling, particularly when done with solely malicious intent as opposed to the motivation of correcting an injustice. Trolling, in other words, can be used as a tool, and this could affect perceived trolling and toxicity rates on different platforms as well. Twitter is an example of a highly political space in which trolling is, according to our participants, rampant. At least a portion of this is due to politically backed actors and bots trolling with a particular ideology in mind (Kargar and Rauchfleisch, 2019; McCombie et al., 2020). A particularly terrifying version of this goes by the name of “chan cultures”; originating from sites such as 4chan and 8kun, these are internet subcultures in which “violence is both trivialized and glorified” (Crawford et al., 2021, p. 982). According to Crawford et al. (2021), this subculture has been connected to far-right groups and offline terrorism through their use of humorous trolling, particularly through spreading memes – a common theme in our own results. All of this goes to show that there is a big world of trolling out there, and our study only begins to uncover the full picture of trolling on both gaming and social media platforms. That being said, it still has some interesting theoretical and practical implications for the future study of trolls, be they political, playful, or otherwise.

### Theoretical and practical implications

The first and most obvious theoretical implication of this work is the importance of the platform. As noted earlier, trolling is an inherently interdisciplinary topic with myriad available definitions for researchers to choose from (see Cook et al., 2021). To complicate this matter further, the media does not stick to a single understanding of what trolling is either, using it at times as a synonym for toxicity (see Conditt, 2020) and at times as a way to describe annoying but largely benign online behaviors akin to pranking in the offline world (see Piedra, 2018). The present work’s findings would suggest that an important part of why we seem to have such difficulty agreeing on what exactly constitutes trolling is because people’s understanding of the term is inherently linked to platforms, at least in part, which differ in many ways. Here, we divide the platform up by primary purpose: entertainment for online games and social networking for social media sites, though we investigated a particular subculture when we did so (game players). However, these are not the only distinctions that can be made between platforms. Gandolfi and Ferdig (2022) have already begun to look at the importance of specific affordances for toxicity; our findings would suggest that future work could take a similar approach, but instead of looking at affordances in a single game, multiple games or social media platforms could be categorized by affordances and their communities investigated to see how toxicity levels and trolling behaviors differ. Avatars, for instance, are a common difference between popular social media (e.g., Instagram) and games (e.g., World of Warcraft).

Another important distinction to make when reflecting upon differences between platforms is the communities that use said platforms. As previously mentioned, we specifically looked at game players in the present work, but even this is a broad categorization. One could imagine that players of World of Warcraft, a fantasy MMORPG, may differ significantly in their usage of their platform than players of the Call of Duty franchise, an intensely competitive first-person shooter. Naturally, these platforms differ in terms of affordances – they are different genres of game – but they are also likely to differ in terms of the social norms of their communities (for a complete discussion of gaming communities, see Mäyrä, 2016). Like games, neither are social media platforms completely homogenous in terms of their communities’ social norms and/or practices (see Paris et al., 2012; Freelon et al., 2018). In short, the present study’s division of platforms is by no means the only option, and future work should explore differences between all kinds of communities across a variety of platforms to help determine the exact mechanisms behind the differences we have observed by talking to trolling targets. By continuing to investigate these fine-grained differences, we may be able to develop a series of individual understandings of trolling that are platform or community specific, better serving policy-makers and researchers alike.

A second important note is that this is not the first time that boredom and frustration have shown up as major motivators for trolling behavior (Thacker and Griffiths, 2012; Cook et al., 2018); it has been years since the original articles talking about these motivations emerged, and yet still today these are coming up in interviews as major trolling catalysts. It is, however, the first time that we have seen this distinction with social media trolling being primarily motivated by boredom and games by frustration. For games, theory would suggest that boredom has something to do with game mastery (see Cook, 2019); when the game no longer holds a challenge, people will find other ways to entertain themselves. Frustration, on the other hand, comes from the opposite end of the spectrum, with the game’s difficulty level exceeding the player’s skill level by too high a margin (Cook, 2019). However, similar theoretical work has yet to be done outside of games. Existing theoretical work on trolling on social media has heavily focused on personality theory to explain trolling behavior in these contexts (e.g., Buckels et al., 2014, 2019; March, 2019), while other studies provide more descriptive information (see Sanfilippo et al., 2018; Sun and Fichman, 2018). Given our findings suggesting that these motivations go beyond games and apply to trolling on social media as well, we need to begin looking at beyond-platform solutions to this issue, interventions that teach young adults to better manage emotions such as boredom and frustration, perhaps. Such interventions have already been designed and implemented with success in various workplaces (see Scott and Myers, 2005; Little et al., 2013); our findings would suggest that it may be worth adapting them for more general use and seeing how they impact trolling rates.

Finally, the present work has highlighted just how much behavioral trolling, or non-verbal trolling, is perceived as a problem in the current gaming landscape. A vast majority of our participants experienced some form of behavioral trolling or at the very least mentioned it as a type of trolling that they see frequently in-game. Participants also indirectly highlighted the perceived reason for behavioral trolling’s prominence in games: an over-reliance on automated content moderation on gaming platforms. Chat filters and the like are, according to our participants, being used *instead* of human moderation teams as opposed to *a support for* human moderation teams. As previously mentioned, trolling is only truly limited by programming and human creativity, and it seems as though behavioral trolling is ingenuity’s current bypass of automatic content moderation techniques. User-based moderation works for websites such as Reddit (2022) and was seemingly effective for a time in League of Legends during the days of the Tribunal (GameFAQs, 2015). Urban legends of games that created special servers for those who trolled excessively exist as well, although these rumors are not fully substantiated (Cook et al., 2018). It is also possible that companies are engaging in antisocial design practices (see Carmi, 2022), in other words, permitting trolling for the purpose of creating outrage, subsequently leading to further engagement (see Spring et al., 2018). However, if this is indeed the case, it would seem as though it only works up to a point as one of the most popular ways noted in the present work to deal with trolling was to stop playing the game, either temporarily or permanently. To avoid player dropout due to excessive behavioral trolling, new methods of moderation should be investigated by both academics and in-house researchers to determine the best balance between player freedom and player protection when it comes to non-verbal trolling.

### Limitations and future directions

Though the present study highlights important differences between perceived trolling on social media and in online games, it is not without its limitations. As previously noted, because this is part of a larger study on game players, almost the entire population uses games more heavily than social media platforms; the study should therefore be replicated on more casual game players to see which trends hold and which are an artefact of our sample. This sampling also meant that our participants were mostly men, while this is not true of internet users taken as a whole. Future work should aim for a more even gender distribution to better capture a wider experience. In addition, several of our participants mentioned important cultural dimensions of trolling but not in enough detail or across enough participants to examine in more depth. Though there have been cross-cultural studies of trolling (see Cook et al., 2021), these have not been done qualitatively across two or more specific cultures. The present study suggests that there are indeed more cross-cultural differences to explore in depth. We were also restricted by our IRB review from asking more questions about gaming and demographic background as the interview protocol was already lengthy, and the reviewers wanted to keep participants as anonymous as possible. In addition, due to the need for coders to be both fluent English users and familiar with a wide variety of games, two of the authors had to be coders; in an ideal world, the coders would have been separate from the authorial team. Finally, the majority of our participants focused on team-based games. It is possible this is because team-based games have more trolling, but it could also be an artifact of our recruiting measures. Future work should aim for a more balanced distribution across gaming genres to potentially find new patterns.

There is still ample room for further work in this area. For instance, the present work did not focus on the emotional experience of victims, something that could be of particular interest to those designing interventions or counselling programs for trolling victims. This could be addressed in future qualitative work. With the categories of behaviors and definitions identified here, researchers could also ask a larger group of representative game players and social media users about their understanding of trolling in a larger-scale survey to determine which results hold true and which are artifacts of the small sample size. The question of generational influence on trolling also remains (see Cook et al., 2018 for further discussion); this could be of interest for future studies to specifically target teenage or older adult trolls or targets to see how age impacts the understanding of this ever-changing phenomenon.

## Data availability statement

The raw data supporting the conclusions of this article will be made available by the authors, without undue reservation.

## Ethics statement

The studies involving human participants were reviewed and approved by the National Chengchi University Research Ethics Committee. The patients/participants provided their written informed consent to participate in this study.

## Author contributions

CC designed the study, performed all data collection, analyzed all data, and wrote and revised the article. ST reviewed all interview transcripts for accuracy, analyzed all data, and reviewed the article. J-HL provided funding for the project and provided critical revisions prior to submission. All authors contributed to the article and approved the submitted version.

## Funding

This study was supported by grants #111-2811-H-004-006 and #109-2423-H-004 -004 -SS4 from the National Science and Technology Council.

## Conflict of interest

The authors declare that the research was conducted in the absence of any commercial or financial relationships that could be construed as a potential conflict of interest.

## Publisher’s note

All claims expressed in this article are solely those of the authors and do not necessarily represent those of their affiliated organizations, or those of the publisher, the editors and the reviewers. Any product that may be evaluated in this article, or claim that may be made by its manufacturer, is not guaranteed or endorsed by the publisher.
