# Supplementary material for: Comparing shades of darkness: trolling victims’ experiences on social media vs. online gaming
Source: Front Psychol. 2023 Aug 22;14:1163244. doi: 10.3389/fpsyg.2023.1163244 (PMC10478268; doi:10.3389/fpsyg.2023.1163244)
Supplement: Supplementary file 1 [file Data_Sheet_1.DOCX]

**Appendix A – Full Interview Protocol (anonymized)**

Hello, my name is XXXX and I am postdoctoral researcher at XXXXX in XXXXX. I’m conducting a series of studies involving trolling victims and these interviews are part of the first study focusing on gathering the real experiences of real victims. There is almost no formal research completely dedicated to talking to trolling victims specifically, as most of the victim research out there focuses more on other types of online victims, like cybercrime and cyberbullying; this study is designed to help fix that by gathering the opinions of real trolling victims to get a better picture of what is really happening online.

This interview should last approximately one and a half hours, and you will be paid 15 euros for your participation. Because of the nature of the topic, you should be aware that there will be some more personal questions involved. You must also know that these interviews are recorded. However, this recording is audio only and will be stored securely, and personally identifying information like your name will not be included in the interview transcripts. You will remain anonymous throughout. You also have the right to stop the interview at any point; if you choose to do so, the recording will be deleted immediately.

So, knowing that the interviews are recorded and approximately one hour to an hour and a half long, do you consent to continuing with the interview? (*Yes/No*)

- No = Thank you for your time; enjoy your day.
- Yes = Thank you for your participation!

**Basic Demographics**

We’re going to start out with some basic information about you. This is so I can better understand how different people respond to the same questions.

1. What is your age?
2. What are your gender identification and preferred pronouns?
3. Do you identify as a racial minority member in your area?
4. What is your country of origin?
5. In which country are you currently living?
6. What is your highest completed level of education?

**Specific ICT usage statistics**

1. Do you use any social media platforms?
   1. Which ones?
   2. How often do you use (on average) that social media per day? Per week?
2. Are you active on any forums or other apps?
   1. Which ones?
   2. How often are you active (on average) on these per day? Per week?
3. Are you still a gamer?
   1. Which games do you play?
   2. How often do you game (on average) per day? Per week?

**Trolling Knowledge**

Ok, now we’re going to talk about your knowledge and opinions of trolling – what you’ve witnessed happening in the past online.

1. What kind of behaviours would you classify as “trolling”?
   1. (If they only talk about games) How about on social media?
2. Where have you seen trolling happen in the past?
3. How often does it happen?
4. Does the kind of trolling you see change depending on where you see it happen?
5. How about the frequency of trolling? Does that change depending on where you see it happen?
6. Is there anything else you’ve seen that you would consider trolling that you haven’t talked about yet?

**Trolling Experience**

This next group of questions deals with your own trolling experiences. These questions are a little more personal than the previous ones.

1. What kind of trolling have you been subjected to in the past?
2. Please think of a specific time in which you were recently trolled.
   1. What happened?
   2. How did you respond immediately?
   3. What did you feel when it happened (emotionally and physically)?
   4. What did you think when it happened?
   5. What did you do to cope in the short-term?
   6. What did you do to cope in the long-term?
   7. How was this time different than when you’re usually trolled?
3. Are you often trolled? On which platforms?
4. Are there any coping techniques that you’ve used in the past that you found weren’t effective for dealing with a trolling experience?
5. Have you ever trolled yourself?
   1. If yes, what did you do?
   2. Why?

**Trolling Support**

Finally, I’m going to ask you about what you think should and should not be happening when it comes to dealing with trolling.

1. What do you think platforms (including games) are doing well when it comes to dealing with trolling?
2. What do you think platforms (including games) could improve on when it comes to dealing with trolling?
3. Are there any platforms that you think are doing particularly well or poorly compared to others?
4. What do you think people should do when they see someone being victimized by a troll?
5. What support should platforms have available for people who are victimized by trolls on their platform?
6. What other resources should be available for victims of trolls?

That is the end of my questions. The goal of these interviews is to fill a major gap in trolling research: the experience of victims. By doing these interviews – and more studies in the near future – I hope to give policy-makers and platforms the knowledge they need to be able to better protect people from the possible negative consequences of trolling.

As I said at the beginning, the recording of this interview will be stored securely and anonymously. If you have any further questions about this study, you can contact me – I will type my e-mail address into the chat box. If you would prefer to contact me by mail, I will provide that address in the chat box as well. Thank you again for this interesting and informative interview; enjoy the rest of your day.
