# Supplementary material for: Comparing shades of darkness: trolling victims’ experiences on social media vs. online gaming
Source: Front Psychol. 2023 Aug 22;14:1163244. doi: 10.3389/fpsyg.2023.1163244 (PMC10478268; doi:10.3389/fpsyg.2023.1163244)
Supplement: Supplementary file 2 [file Data_Sheet_2.DOCX]

Appendix B – Codebook

- Codes for RQ1 – Definitions of trolling
  - 0 - Didn't mention anything
  - 1 - Intentional Antagonism
  - 2 - Canceling
  - 3 - Misrepresentation
  - 4 - Breach of privacy
  - 5 - Not trying to win
  - 6 - Playful 'messing with'
- Codes for RQ2 – Behaviours associated with trolling on different platforms
  - 1 - Profanity/Flaming
  - 2 - Doxing
  - 3 - Verbal harassment
  - 4 - Misinformation
  - 5 - Provocation
  - 6 - Behavioural trolling
  - 7 - Make fun of or tease
  - 8 - Spamming
  - 9 – Lying*
  - 10 - Personal insults
  - 11 - Hacking
  - 12 – Memes
- Codes for RQ3 – Reasons behind toxicity levels on various platforms
  - 1 - Company policies
  - 2 - Content moderation
  - 3 - Moderation tools
  - 4 - Community norms
  - 5 – Bots**
  - 6 - Competition (or lack thereof)
  - 7 - Racism/Sexism
  - 8 - Anonymity
  - 9 - None given
  - 10 – Affordances

* Was eventually merged into misinformation after discussion between coders.

** Was originally a separate code, but was merged into moderation tools after discussion between coders.
